# The Gut Microbiota in Women Suffering from Gestational Diabetes Mellitus with the Failure of Glycemic Control by Lifestyle Modification

**DOI:** 10.1155/2019/6081248

**Published:** 2019-10-23

**Authors:** Guangyong Ye, Long Zhang, Min Wang, Yunbo Chen, Silan Gu, Keyi Wang, Jianhang Leng, Yujia Gu, Xinyou Xie

**Affiliations:** ^1^Women's Hospital, Zhejiang University School of Medicine, China; ^2^The First Affiliated Hospital, Zhejiang University School of Medicine, China; ^3^Central Laboratory, Affiliated Hangzhou First People's Hospital, Zhejiang University School of Medicine, China; ^4^Department of Clinical Laboratory, Sir Run Run Shaw Hospital, Zhejiang University School of Medicine, China

## Abstract

Gestational diabetes mellitus (GDM) is prevalent worldwide, leading to a high risk of significant morbidity for both the mother and offspring with complications. Increasing evidences suggest that gut microbiota plays a role in the pathogenesis of GDM. Lifestyle modification is the cornerstones of GDM treatment. However, a number of patients whose blood glucose is not controlled by lifestyle modification still require exogenous insulin to control blood glucose. No observational study is available about the relationship between the gut microbiota in GDM patients and lifestyle modifications. Thus, we investigated the differences in gut microbiota between GDM patients with successful glycemic control (GDM1) and failure of glycemic control (GDM2) by lifestyle modifications. We sequenced the V3-V4 regions of 16S ribosomal ribonucleic acid (rRNA) gene from stool samples of 52 singleton pregnant women with 24–28 weeks of gestation. Our results showed that *Blautia*, *Eubacterium_hallii_group*, and *Faecalibacterium* in the gut microbiota showed significant differences among the normoglycemic mother, GDM1, and GDM2 groups, respectively. The combined diagnostic performance of *Blautia*, *Eubacterium_hallii_group*, and *Faecalibacterium* in differentiating GDM2 from GDM was considered as the most reasonable identification indicator. Gut bacteria may participate in the pathological development of GDM2 through the peroxisome proliferator-activated receptor (PPAR) signaling pathway. These results indicated that *Blautia*, *Eubacterium_hallii_group*, and *Faecalibacterium* had important characteristic changes in the gut microbiota of women with GDM2.

## 1. Introduction

Gestational diabetes mellitus (GDM) is defined as glucose intolerance or hyperglycemia of varying severity with the onset or first recognition during pregnancy [1]. During pregnancy, the mother's body undergoes a series of physiological changes (i.e., metabolic adaptation) in order to support the demands of the growing fetus. During early gestation, insulin sensitivity increases, promoting the uptake of glucose into adipose stores in preparation for the energy demands of later pregnancy. In the second and third trimesters of pregnancy, the antagonism of insulin-like substances leads to decreased insulin sensitivity in pregnant women. The demand for insulin increases accordingly to maintain normal blood glucose levels. However, the normal insulin levels during pregnancy do not adequately occur in all pregnancies, except in those with preexisting insulin resistance or women who cannot increase insulin secretion, resulting in GDM [2, 3].

The prevalence of GDM is very high worldwide [4, 5], and in some countries or areas, it is even higher than 20% [6]. The incidence of GDM increases continuously, leading to a severe morbidity for both mother and offspring with short-term complications such as preeclampsia [7], macrosomia [7, 8], cesarean delivery [8], neonatal hypoglycemia [9], and congenital malformation [10], while long-term complications included maternal type 2 diabetes mellitus and cardiovascular diseases, as well as obesity, diabetes mellitus, and other metabolic diseases in the offspring [11–15]. Lifestyle modifications that focus on changes in diet and physical activity act as a cornerstone and first choice of treatment for GDM [16]. Till date, the blood glucose levels can be controlled by lifestyle modifications in approximately two-thirds of patients with GDM [17]. However, majority of patients whose blood glucose cannot be controlled by lifestyle modifications require exogenous insulin to control blood glucose levels [18].

The gut microbiota contributes to the pathogenesis of diabetes, and the relationship between these is an important topic of clinical concern and research [19, 20]. There is an emerging evidence that the gut microbial organisms—the “gut microbiome”—might contribute to metabolic diseases, including GDM [2, 21–23]. Studies on the gut microbiota of GDM have showed contrast results, either no differences [24], with increased abundance of Firmicutes and reduced Bacteroidetes and Actinobacteria [25] or decreased abundance of the *Faecalibacterium* reported in comparison with normoglycemic mothers [26]. However, another article reported opposite results regarding the abundance of *Faecalibacterium* in GDM [27]. Additionally, some studies have addressed the relationship between the gut microbiota and the development of GDM [27–29]. Till date, there are very few observational studies available about the relationship between the gut microbiota in GDM patients and lifestyle modifications.

## 2. Materials and Methods

### 2.1. Study Population

From September 1, 2016 to November 28, 2017, 52 singleton pregnant women during 24-28 gestational weeks were recruited from the outpatient department of the Women's Hospital, Zhejiang University School of Medicine. Of these, 36 women were with GDM and were further divided into successful glycemic control (GDM1 group, *n* = 24) and failure of glycemic control (GDM2 group, *n* = 12) based on the results of glycemic control after lifestyle modifications. The remaining 16 were normoglycemic pregnant women (N group).

The exclusion criteria were as follows: pregnant women with prepregnancy diabetes mellitus, family history of diabetes mellitus, hypertension, Cushing syndrome, hyperthyroidism, hyperlipidemia, genetic disease, and other diseases that affect glucose metabolism and pregnant women with inflammatory bowel disease, irritable bowel syndrome, and celiac disease, using antibiotic or probiotics within one month that affect the gut microbiota were excluded. Pregnant women who did not comply with the principles of the trial and medication were also excluded from this study. All the study procedures met the ethical guidelines of the World Medical Association Helsinki Declaration 2013. All participants provided a written informed consent form. The study was approved by the Ethics Committees of the Women's Hospital, Zhejiang University School of Medicine (approval number: protocol #20160081).

### 2.2. Data Collection and Specimen Collection

The oral glucose tolerance test (OGTT) was conducted in women with 24-48 weeks of gestation according to the diagnostic criteria of GDM for diagnosing GDM. Participants were asked to fast for at least 8 h prior to each clinic visit and avoid smoking and heavy physical activity for the preceding 2 h. After that, 3.0 mL venous blood was drawn on empty stomach from pregnant women and underwent centrifugation at 3000 rpm for 5 minutes. The serum was separated and immediately sent for examinations, such as fasting blood glucose (FBG), total cholesterol (TC), triglyceride (TG), low-density lipoprotein cholesterol (LDL-C), and high-density lipoprotein cholesterol (HDL-C). Then, 300 mL aqueous solution containing 75 g of glucose was taken, and blood was drawn at 1 h and 2 h, respectively.

On the same day, their stool was collected. All pregnant women in our study were asked to put their stool in a clean presupplied container by staff and submit the stool for examination within 1 h. Our researchers then used a sterile spoon to scoop 3-5 grams of fresh stool from the center of the whole homogenate stool and placed it in a freezer at −80°C within 2 hours of collection.

The participants with GDM then attended a 2 h group counseling session led by nutritionists and nurses in our GDM follow-up center and were subsequently recommended for lifestyle modifications. Meanwhile, the basic characteristic information of the participants was collected. Body weight was measured by trained and certified technicians to the nearest 0.1 kg using a calibrated scale by requesting participants to wear light clothing. Height (without shoes) was measured to the nearest 0.1 cm using a vertical ruler. Body mass index (BMI) was calculated by dividing weight in kilograms by height in meters squared. Blood pressure (BP) was measured after the participants were seated and rested for 5 min by using an Omron aneroid device. To determine the waist circumference, the nurse locates the upper hip bone and places a measuring tape around the abdomen. After 3 to 4 weeks, these participants were informed to visit the clinic again for blood glucose measurement according to the goal of glycemic control during pregnancy.

### 2.3. Diagnostic Criteria for GDM and the Goal of Glycemic Control for GDM

The diagnosis of GDM was made if any of the following blood glucose values are met: (1) FBG ≥ 5.1 mmol/L, (2) 1-hour blood glucose post 75 g oral glucose load ≥ 10.0 mmol/L, and (3) 2-hour blood glucose post 75 g oral glucose load ≥ 8.5 mmol/L [30].

The goal of glycemic control for GDM after lifestyle modifications was as follows: FBG < 5.3 mmol/L and either 1-hour blood glucose post 75 g oral glucose load < 7.8 mmol/L or 2-hour blood glucose post 75 g oral glucose load < 6.7 mmol/L [30]. If the above criteria are met, these pregnant women are classified under the GDM1 group, and if not, they are classified under the GDM2 group. The women in the latter group were treated with insulin or hypoglycemic drugs to achieve acceptable glycemic levels.

### 2.4. Lifestyle Modification

Lifestyle modifications involve dietary therapy and physical activity. Routine follow-up and diet adjustments were provided throughout pregnancy to achieve and maintain the goal of glycemic levels. Training, education, support, and follow-up (telephone) by a qualified doctor who is experienced in taking care of women with GDM were also provided. Dietary intake and physical activities were assessed by an interviewer-administered questionnaire.

A goal for the required energy intake from the diet should be obtained for each participant. Diet and energy intake were calculated based on the weight and blood glucose levels. The total energy intake was within the scope of ideal body weight multiplied by energy coefficient taken away from 35 kcal/kg per day. In addition, limiting the daily energy intake to 1800–2000 kcal is considered appropriate (Tables [Supplementary-material supplementary-material-1] and [Supplementary-material supplementary-material-1]). If the blood glucose levels of GDM women who can control the daily energy intake to 2000 kcal after dietary therapy, then it will be suitable for them to ingest 2000 kcal energy per day. Otherwise, they are recommended to reduce their daily energy intake to 1800 kcal.

Each participant followed a specific exercise program. The forms of exercise included continuous moderate-intensity exercise that mainly involves the large muscles of the body, such as walking, upper limb strength training exercise, and jogging. In addition, it was better to perform the same intensity physical activity at the same time each day. Our doctors train the family members on how to use the glucometer. Self-monitoring of blood glucose for women with GDM should be carried out every day.

### 2.5. Detection of Biochemical Indicators

Blood glucose was measured by the glucose oxidase-peroxidase method [31]. The main principle including: the enzyme glucose oxidase catalyzes the oxidation of glucose to gluconic acid and hydrogen peroxide (H_2_O_2_). Addition of the enzyme peroxidase and a chromogenic oxygen acceptor results in the formation of a colored compound that is measured. TC was measured by the cholesterol oxidase (CHOD) phenol 4-aminoantipyrine peroxidase (CHOD-PAP) method [31]. The main principle including: the reagents typically use a bacterial cholesteryl ester hydrolase to cleave cholesteryl esters (CHE). The 3-OH group of CHE is then oxidized to a ketone by CHOD. H_2_O_2_, one of the reaction products, is measured in a peroxidase-catalyzed reaction that forms a colored dye. TG was measured by the glycerol phosphate oxidase phenol 4-aminoantipyrine peroxidase (GPO-PAP) method [31]. The main principle including: lipase catalyzes the hydrolysis of TG to glycerol and fatty acids. Glycerol then is phosphorylated by glycerokinase. Glycerophosphate is then oxidized to dihydroxyacetone and H_2_O_2_ in a glycerophosphate oxidase-catalyzed reaction, and the H_2_O_2_ formed in the reaction is measured. LDL-C was measured by a surfactant method [31, 32]. The main principle including: polyanions and amphoteric surfactants were selectively protected LDL from enzymatic reaction. The non-LDL CHE reacted with esterase and oxidase, producing H_2_O_2_, which was consumed by catalase. 4-Aminoantipyrine, peroxidase, and a deprotecting reagent could remove the protecting agent from LDL, enabling the specific reaction of CHE and CHOD with its CHE, producing H_2_O_2_ and a color complex. And HDL-C was measured by the peroxidase-scavenging method [31, 32]. The main principle including: CHE and CHOD were allowed to react with lipoproteins other than HDL, generating peroxidase, which in turn was scavenged by the enzyme catalase. An inhibitor of catalase and a surfactant in a second reagent specifically reacted with HDL-C, producing color through the usual peroxidase sequence. All the above reagents are purchased from Biosino Bio-Technology, China. All the above items are determined by an ABBOTT ARCHITECT c1600 automatic biochemical analyzer (ABBOTT, USA), following the manufacturer's instructions.

### 2.6. Deoxyribonucleic Acid (DNA) Extraction

Stool samples (200 mg) were processed by using a grinding bead homogenizer (FastPrep; Thermo Electron Corporation, MA, USA) and used for DNA extraction with a QIAamp DNA Stool Mini Kit (Qiagen, Hilden, Germany) according to the manufacturer's protocol. The DNA content was measured using a NanoDrop ND-100 spectrophotometer (NanoDrop Technologies, DE, USA). The DNA integrity and size were measured by electrophoresis on a 1.0% agarose gel containing 0.5 mg/mL ethidium bromide. The extracted DNA was stored at −20°C for further analysis. A negative control was performed during DNA extraction. The specimens were treated separately in the biosafety cabinet. The experimental clothes and disposable gloves were used, and the liquid pipette gun head with filter membrane, disposable reaction tube, and centrifugal tube were used. Cross-use of equipment in different areas of the laboratory is strictly prohibited.

### 2.7. 16S V3-V4 Region Amplification, Product Purification, and Sequencing

Universal primers 338F (5′-NNNNNNNNACTCCTACGGGAGGCAGCA-3′) and 806R (5′-NNNNNNNNGGACTACHVGGGTWTCTAAT-3′) for bacterial 16S V3-V4 were used for polymerase chain reaction (PCR) amplification to obtain V3-V4 region sequence of the bacterial 16S ribonucleic acid (rRNA) gene. NNNNNNNN that is added to the 5′ end of the upstream and downstream primers is an 8-bit tag base sequence (barcode) obtained for each sample and used to distinguish between samples during sequencing. Each sample was processed thrice. The PCR products of the same sample were then mixed and detected using 2% agarose gel electrophoresis. PCR products were recovered using the AxyPrep DNA Gel Recovery Kit (Axygen, Union City of California, USA), eluted with Tris-HCl, and detected on a 2% agarose gel. Based on the initial quantitative results of electrophoresis, PCR products were quantified using a QuantiFluor-ST Blue Fluorescence Quantitation system (Promega, Madison, WI). The PCR products of 52 samples were mixed in equimolar amounts according to the measured concentrations and sequenced on an Illumina MiSeq platform as required.

A negative control was performed during the PCR amplification process. When the negative control test is negative, it shows that the reagent in the whole process of the test is not contaminated. Strategies to prevent cross-contamination: special operation areas are set up, and different work areas are separated from each other and transmitted through transfer warehouses. Different experimental steps were operated in a continuous and independent space. Cross-use of equipment in different areas of the laboratory is strictly prohibited. The laboratory was disinfected by ultraviolet radiation regularly. All reagents or equipment should be sterilized under high pressure, except enzymes and substances that cannot withstand high temperature.

### 2.8. Sequencing Data Processing, Operational Taxonomic Unit Analysis, and Species Annotation

Paired-end reads obtained from MiSeq sequencing were initially put together using flash software according to the overlapping relationship. Quality control was performed based on barcode and primer sequences in order to obtain a valid sequence. Quantitative Insights into Microbial Ecology (QIIME) software was used for sequencing data filtration by removing low-quality bases and contaminated sequences for obtaining a high-quality target sequence for subsequent analysis [33, 34]. The sequences were clustered into “operational taxonomic units” (OTUs) based on 97% similarity with USEARCH software (version 7.0 http://drive5.com/uparse/) [35]. During this process, chimeras were removed using the UCHIME algorithm [36], and finally, the representative sequence of OTU was obtained. A set of sequences that belonged to the same OTU were considered related. Among this set, one representative sequence was selected for species annotation using the Ribosomal Database Project (RDP) Classifier [37, 38]. SILVA (Release128 http://www.arb-silva.de) database was used to classify these sequences into specific taxa [39].

### 2.9. Imputed Metagenomic Analysis

The metagenomes of gut microbiome were imputed from 16S rRNA sequences with PICRUSt (Phylogenetic Investigation of Communities by Reconstruction of Unobserved States) [40]. This method predicts the gene family abundance from the phylogenetic information with an estimated accuracy of 0.8. For every individual and Kyoto Encyclopedia of Genes and Genomes (KEGG) pathway, PICRUSt estimates the total gene count within that pathway (normalized to a relative abundance per pathway). The closed OTU table was used as the input for metagenome imputation and was first rarefied to an even sequencing depth prior to the PICRUSt analysis. Next, the resulting OTU table was normalized by 16S rRNA gene copy number. The gene content was predicted for each individual. Then, the predicted functional composition profiles were collapsed into level 3 of KEGG database pathways [40, 41].

### 2.10. Linear Discriminant Analysis (LDA) Coupled with Effect Size (LEfSe)

LEfSe is software for discovering high-dimensional biomarkers and revealing genomic characteristics, including genes, metabolism, and classification, to distinguish two or more biological conditions (or groups) [42]. LDA of LEfSe analysis include three main steps: first, the Kruskal-Wallis test was used to analyze all the characteristic species, to detect the species abundance differences among different groups, and to obtain significantly different species. Then, the Wilcoxon test was used to check whether all subspecies in the species with significant differences converged to the same taxonomic level. Finally, a LDA model is established for the generated vector set, and the list of different species and the effect size at a specific taxonomic level are obtained. In our study, LDA of LEfSe was coupled with to search for statistically different biomarkers between groups using the Kruskal-Wallis test (*P* < 0.05) with LDA score > 4.0. The LDA score was obtained by LDA (linear regression analysis); the larger the LDA score, the greater the influence of species abundance on the difference effect.

### 2.11. Statistical Analysis

Mothur was used to generate rarefaction data [43]. Venn diagram was generated with a VennDiagram R package (http://www.r-project.org) [44]. Alpha diversity metrics (Sobs, Chao, Shannon, Simpson, Shannoneven, and Simpsoneven index) and beta diversity (principal coordinates analysis (PCoA analysis)) were calculated with the phyloseq R package [45, 46]. The Shapiro-Wilk test was used to judge the normal distribution of data [47]. Measurement data with normal distribution were described as $\overset{¯}{X}\pm \text{SD}$. The differences in blood glucose, age, BMI, and lipid-related parameters among the three groups were analyzed by analysis of variance (ANOVA). Potential trends in blood glucose levels among the three groups were explored using the ANOVA-trend analysis test. The index microbiota diversity and richness and relative abundance of the bacteria among the three groups were compared with the Kruskal-Wallis *H* test [48], and the nonparametric test in pairwise comparison of multiple groups was the Dunn-Bonferroni test (*P* values were corrected by the Benjamini-Hochberg method) [49, 50]. Correlation analysis was performed by Spearman's rank correlation test. Receiver operating characteristic (ROC) curve was used to evaluate the diagnostic efficiency of major bacteria. Statistical Product and Service Solutions (SPSS) software (version 22.0; SPSS, Inc., IL, USA) and GraphPad Prism software (version 7.0; GraphPad Software, Inc., San Diego, CA) were used for data analysis and generate the figures. All tests were two sided, and *P* < 0.05 was considered as statistically significant.

## 3. Results

### 3.1. General Participant Information

The average age of all pregnant women was 33 years, and their gestational age was 24.6 weeks, with a BMI of 25. No differences in age (*F* = 2.8, *P* = 0.07), gestational age (*F* = 2.1, *P* = 0.14), body weight (*F* = 1.8, *P* = 0.18), height (*F* = 0.08, *P* = 0.93), and BMI (*F* = 2.5, *P* = 0.09) were observed among the N, the GDM1, and the GDM2 groups. However, the differences in FBG (*F* = 49.9, *P* < 0.001), 1-hour blood glucose post 75 g oral glucose load (*F* = 37.8, *P* < 0.001), and 2-hour blood glucose post 75 g oral glucose load (*F* = 32.5, *P* < 0.001) showed significant differences among these three groups. FBG (*F* = 92.57, *P* = 7.14*E*‐13), 1-hour blood glucose post 75 g oral glucose load (*F* = 67.06, *P* = 9.83*E*‐11), and 2-hour blood glucose post 75 g oral glucose load (*F* = 55.36, *P* = 1.38*E*‐9) showed an upward trend among the N, the GDM1, and the GDM2 groups by the ANOVA-Trend Analysis test, respectively. Also, the TC, HDL-C, and LDL-C levels showed significant differences (*P* < 0.05) (Table 1).

### 3.2. OTU Sequence Diversity and Richness

Sequencing of the V3 region of 16S rRNA gene was performed on stool samples. After optimizing the sequence information, 2,054,594 high-quality sequences were obtained, with an average of 39,511 sequences per sample (30,024–58,559) and 432 bp (426-443) per sequence, which were used for subsequent data analysis. The rarefaction curve tended to be flat, indicating that the amount of sequencing data was large enough, and the amount of sequencing data was reasonable. This suggested the presence of vast majority of microbial diversity information in the samples (Fig. [Supplementary-material supplementary-material-1]). The sequencing data were more appropriate, and the sequencing depth already covered most of the species in the sample. All samples were downsampled to contain 10,000 sequences to compare the diversity parameters by avoiding the influence of the total number on sample sequences.

**Figure 1:**
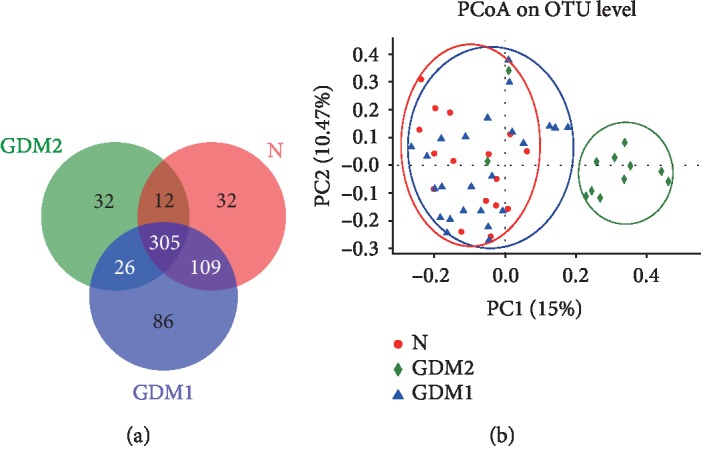
Comparison of gut microflora composition among the N, the GDM1, and the GDM2 groups. (a) Venn diagram showing the overlap of OTUs. (b) Based on the Spearman coefficient, PCoA was used to evaluate the differences in the fecal bacteria of all groups.

Furthermore, 602 reference OTUs from 52 stool samples belonged to 218 genera out of 13 different phyla. The observed species richness (Sobs) and estimated richness for Chao 1 (Chao) showed no differences among the three groups, indicating no difference in microbiota community richness. The Shannon diversity index (Shannon) and the Simpson diversity index (Simpson) showed no differences among the N, the GDM1, and the GDM2 groups, indicating no difference in microbiota community diversity among the three groups. Also, the Simpson index-based measure of evenness (Simpsoneven) and the Shannon index-based measure of evenness (Shannoneven) showed no differences among the N, the GDM1, and the GDM2 groups, indicating no difference in microbiota community evenness among the three groups ([Supplementary-material supplementary-material-1]). All sequences were divided into 602 OTUs according to 97% similarity, and the Venn diagrams that visually represented the total and unique conditions of the OTU numbers of the three groups were formed (Figure 1(a)). The OTU numbers of the N, the GDM1, and the GDM2 groups were 458, 526, and 375, respectively. More than 50% of the OTUs were simultaneously shared by the three groups. The N group shared 414 OTUs with the GDM1 group. Based on the Spearman coefficient, PCoA main coordinate analysis was performed, which showed that the GDM1 and the GDM2 groups were clustered and well distinguished. The N and the GDM1 groups were more clustered and could not be effectively distinguished (Figure 1(b)).

### 3.3. Gut Microbiota Differences between the N Group and the GDM1 Group or the GDM2 Group

At the phylum, class, order, and family levels, the relative abundance of gut microbiota was observed in the N group, the GDM1 group, and the GDM2 group (Fig. [Supplementary-material supplementary-material-1]).

With the Kruskal-Wallis *H* test, the relative abundance of the genus level revealed that *Blautia* (*P* = 2.791*E*‐4), *Faecalibacterium* (*P* = 2.968*E*‐3), *Eubacterium_hallii_group* (*P* = 4.270E‐5), *Subdoligranulum* (*P* = 0.016), *Phascolarctobacterium* (*P* = 0.026), and *Roseburia* (*P* = 0.033) showed significant differences among the N, the GDM1, and the GDM2 groups ([Supplementary-material supplementary-material-1]).

**Figure 2:**
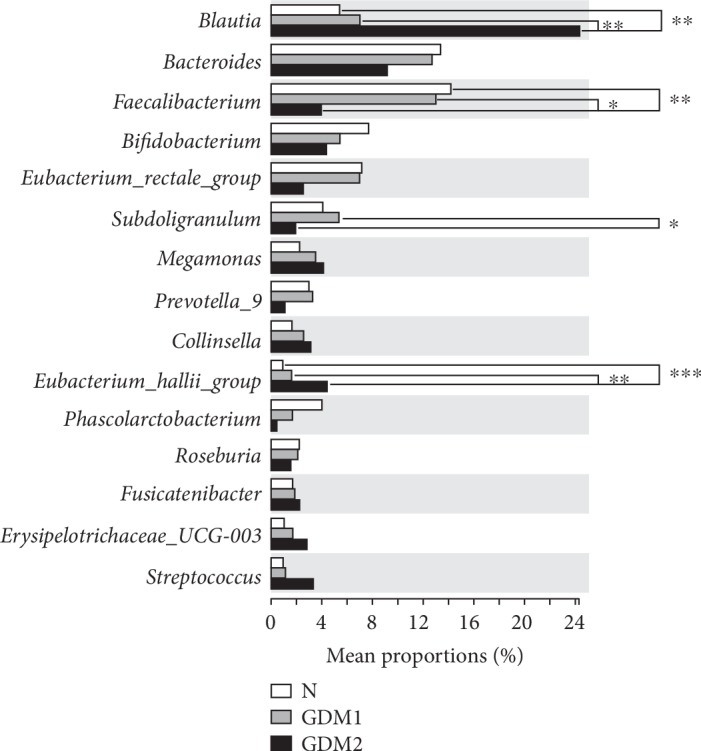
Gut microbiota differences among the N, the GDM1, and the GDM2 groups. Pairwise comparison in multiple groups was conducted with the Dunn-Bonferroni test (*P* values were corrected by the Benjamini-Hochberg method): ^∗^*P* < 0.05, ^∗∗^*P* < 0.01, and ^∗∗∗^*P* < 0.001.

We further compared the relative abundance of the above six bacteria by pairwise comparison with multiple groups of nonparametric test including the Dunn-Bonferroni test (*P* values were corrected by the Benjamini-Hochberg method). The results showed no significant difference between the N group and the GDM1 group. Compared with the N group and the GDM1 group, the relative abundance of *Faecalibacterium* and *Subdoligranulum* in the GDM2 group was lower, while that of *Blautia* and *Eubacterium_hallii*_*group* was significantly higher (Figure 2). Next, Spearman's rank correlation test was used for analyzing potential trends, and the results showed gradual increase of *Blautia* (*r* = 0.533, *P* = 4.8*E*‐5) and *Eubacterium_hallii_group* (*r* = 0.599, *P* = 3.0*E*‐6) in the order of N, GDM1, and GDM2, respectively, while *Faecalibacterium* (*r* = ‐0.419, *P* = 0.002) showed opposite results, but the remaining bacteria showed no correlation ([Supplementary-material supplementary-material-1]).

Therefore, our results indicated the existence of different characteristics of gut microbiota between GDM patients with success and failure of glycemic control by lifestyle modifications.

### 3.4. Correlation between Gut Bacteria and Blood Glucose Levels

**Figure 3:**
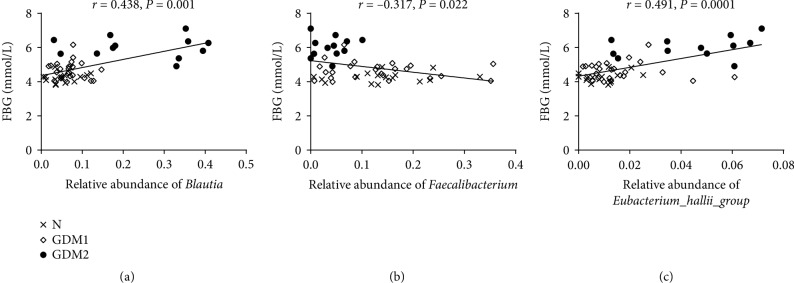
The correlation analysis between the relative abundance of *Blautia*, *Faecalibacterium*, *Eubacterium_hallii_group*, and blood glucose levels.

To understand the close relationship between gut bacteria and blood glucose metabolism, the correlation between relative abundance of *Blautia*, *Eubacterium_hallii_group*, *Faecalibacterium*, *Phascolarctobacterium*, *Subdoligranulum*, *Roseburia*, and FBG was analyzed, respectively. The relative abundance of *Blautia* (*r* = 0.438, *P* = 0.001) and *Eubacterium_hallii_group* (*r* = 0.491, *P* = 0.0001) showed positive correlation with FBG. The relative abundance of *Faecalibacterium* (*r* = ‐0.317, *P* = 0.022) was negatively correlated with FBG. No other bacteria showed correlation with FBG (Figure 3).

### 3.5. Clinical Value of Gut Bacteria in GDM

**Figure 4:**
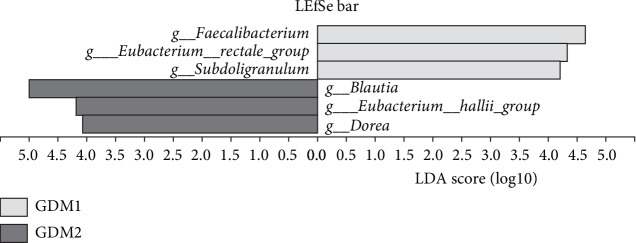
Taxonomic biomarkers between GDM1 and GDM2.

To find biomarkers for differentiating GDM2 from GDM, LDA of LEfSe was used to determine the taxa that explain the differences between the GDM1 group and the GDM2 group. At genus level, 6 species had LDA scores > 4.0. The taxa were compared to obtain the best biomarkers that can predict each category. The results revealed that *Faecalibacterium, Blautia*, and *Eubacterium_hallii_group* among all the bacteria showed significant discriminatory function between the GDM1 group and the GDM2 group (Figure 4). Meanwhile, *Faecalibacterium*, *Blautia*, and *Eubacterium_hallii_group* among all the bacteria demonstrated significant discriminatory function between the N group and the GDM2 group (Fig. [Supplementary-material supplementary-material-1]), but not between the N group and the GDM1 group.

**Figure 5:**
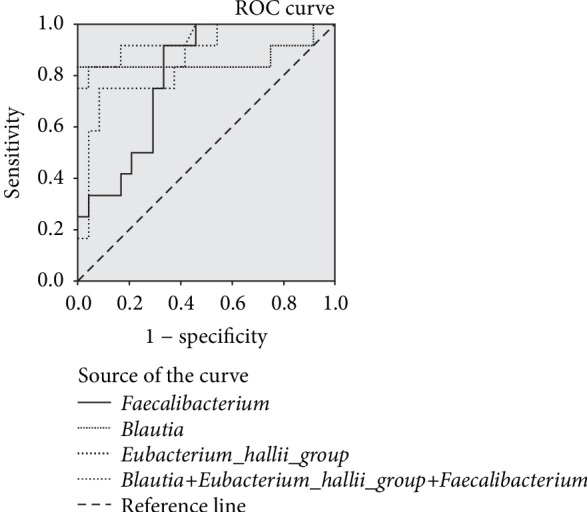
Fitting ROC curve of *Blautia*, *Eubacterium_hallii_group*, and *Faecalibacterium* in the differential diagnosis of GDM1 and GDM2.

To further analyze the sensitivity and specificity of the above three bacteria in the differential diagnosis of GDM1 and GDM2, the ROC curves were fitted to analyze the relative abundance of these three bacteria. The area under the ROC curves (AUC) of *Faecalibacterium* was 0.80, and when the best cut-off point for relative abundance diagnosis was 7.42%, the sensitivity and specificity of GDM2 were 0.92 and 0.67, respectively. The AUC of *Blautia* was 0.86, and when the best cut-off point was 13.18%, the sensitivity and specificity of GDM2 were 0.83 and 0.96, respectively. The AUC of *Eubacterium_hallii_group* was 0.87, and when the optimal cut-off point was 3.37%, the sensitivity and specificity of GDM2 were 0.75 and 0.92, respectively. We analyzed the combined diagnostic performance of *Faecalibacterium*, *Blautia*, and *Eubacterium_hallii_group* in differentiating GDM2 from GDM. The AUC of the combination bacteria was 0.94. At the optimal cut-off point, the sensitivity and specificity of GDM2 were 0.83 and 1.00, respectively (Figure 5).

### 3.6. Microbial Functions Altered during GDM

**Figure 6:**
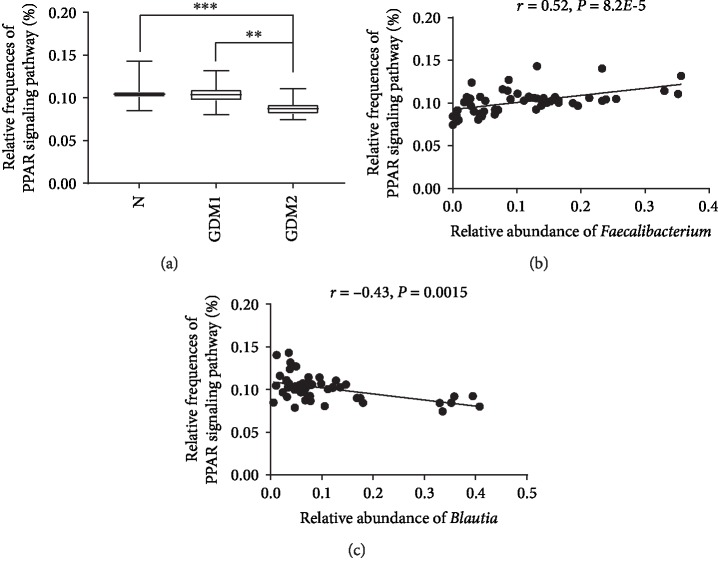
The predicted functional module involving PPAR signaling pathways altered in GDM2. (a) Relative frequencies of the PPAR signaling pathway among N, GDM1, and GDM2; (b, c) correlation between relative frequencies of the PPAR signaling pathway and *Faecalibacterium* or *Blautia* with Spearman's rank correlation test.

To characterise the functional alterations of the gut bacteria in GDM, we predicted the functional composition profiles from 16S rRNA sequencing data with PICRUSt among the N, the GDM1, and the GDM2 groups. We found that multiple KEGG (level 3) categories of the endocrine system (level 2) were disturbed in GDM2. The pathways including the insulin signaling pathway, the peroxisome proliferator-activated receptor (PPAR) signaling pathway, and the adipocytokine signaling pathway, which were deficient in GDM2 patients compared with N and GDM1 (Figure 6(a) and [Supplementary-material supplementary-material-1]). Intriguingly, after correlation analysis of the above signal pathways with *Faecalibacterium*, *Blautia*, and *Eubacterium_hallii_group*, it was found that PPAR was positively correlated with the relative abundance of *Faecalibacterium* (*r* = 0.52, *P* = 8.2*E*‐5) and was negatively correlated with the relative abundance of *Blautia* (*r* = 0.43, *P* = 0.0015) (Figures 6(b) and 6(c)).

## 4. Discussion

In the present gut microbiome study, the association between gut microbiota and GDM status was observed. Specifically, *Blautia* and *Eubacterium_hallii_group* were enriched in the GDM2 groups, whereas *Faecalibacterium*, *Subdoligranulum*, *Phascolarctobacterium*, and *Roseburia* were enriched in the N groups or the GDM1 groups. PCoA main coordinate analysis was performed, and the results revealed that the GDM1 group and the GDM2 group showed clustering and were well distinguished. However, there are no differences in the gut microbe composition and the relative abundance between the GDM1 group and the N group. To our knowledge, this is the first study to explore the characteristics of gut microbiota in GDM with success or failure of glycemic control by lifestyle modifications.

It is interesting to note that *Blautia*, *Faecalibacterium*, *Eubacterium_hallii_group*, and *Subdoligranulum* have shown significant characteristic changes in the gut microbiota of GDM2 patients. *Blautia* and *Eubacterium_hallii_group* were gradually increased in the order of N, GDM1, and GDM2, while *Faecalibacterium* showed opposite results. *Blautia* was presented with enriched abundance in glucose-intolerant individuals [51] and associated with metabolites, reflecting an unhealthy metabolic state in individuals with high BMI [52]. These findings are in line with our study results, showing an increased abundance association with GDM. These results suggested that enriched *Blautia* abundance goes together with a nonfavorable metabolic profile. *Blautia* and *Eubacterium_hallii_group* belonged to Lachnospiraceae, and Lachnospiraceae contributes to the development of diabetes [53, 54]. According to a previous study, there was a reduced abundance of *Faecalibacterium* in women with GDM [26].

In addition, a strong correlation between several discriminatory bacteria and blood glucose levels was observed in our study. It was reported recently [28, 55] that metagenomic linkage groups (MLGs) were obtained by classifying and summarizing the relative abundance of microbial gene sequence markers in fecal specimens; the ratio of gross abundance between GDM-enriched MLGs and control-enriched MLGs was positively correlated with blood glucose levels, which was suggested that microbiome dysbiosis might have a direct association with the pathophysiology of GDM. Similar observations were observed also in healthy young males, where inactivity produced insulin resistance, also in cases devoid of significant community changes. This shows that microbes are capable of adjusting their physiological activities in response to the host signals or host-provided environment [56–58]. We observed that *Blautia*, *Eubacterium_hallii_group*, and *Faecalibacterium* were related to blood glucose levels. *Faecalibacterium* is mainly fermented to produce short-chain fatty acids (SCFA) such as butyrate [59]. When butyric acid is deficient in the gut, then the tricarboxylic acid cycle of colonocytes was inhibited. If butyric acid is added to the colon in time, then it can rescue the defects of mitochondrial respiration and prevent autophagy in colonocytes [60]. If autophagy occurs, then the tight junctions are lost, and the permeability increases between cells. Hence, the absorption of exogenous antigens such as lipopolysaccharides continuously increases, rendering the body in a state of low-grade inflammation by stimulating the production of a large number of inflammatory factors and leading to insulin resistance. In addition, butyric acid works as a histone deacetylase inhibitor [61, 62] that promotes *β*-cell differentiation and proliferation, improving insulin resistance. Lachnospiraceae, including *Blautia* and *Eubacterium_hallii_group*, led to the development of obesity and diabetes by promoting the dysfunction of islet *β-*cells [53, 54]. *Eubacterium_hallii_group* can metabolize glycerol into reuterin [63, 64]. Reuterin can induce oxidative stress by interacting with intracellular glutathione [63, 65]. Oxidative stress can lead to cellular damage by interfering with the state of proteins, lipids, and DNA and has been implicated in the pathogenesis of GDM [66]. It is interesting that *Eubacterium_hallii_group* also produces SCFA [67]. The specific mechanism of SCFA produced by bacteria *Faecalibacterium* and *Eubacterium_hallii_group* in the occurrence and development of GDM needs further study.

To find biomarkers, ROC curve fitting analysis showed that the combination panel of the three bacteria, *Faecalibacterium*, *Blautia*, and *Eubacterium hallii group*, showed a significant improvement in differentiating GDM2 from GDM, reflecting a high clinical value. As the diagnostic index of GDM, OGTT requires prolonged fasting, repeated puncture, and blood sampling, resulting in patient discomfort substantially. Patients must cooperate with the physicians for the entire process. Factors such as severe exercise and mental stress that affects the blood glucose levels are not allowed. However, the detection of gut microbes may provide a promising approach for developing GDM biomarkers, especially for the gut microbes that are very convenient for sampling.

Therefore, we consider *Faecalibacterium*, *Blautia*, and *Eubacterium_hallii_group* as diagnostic markers in women diagnosed with GDM2, i.e., who cannot control the disease by lifestyle modifications.

Analysis of the inferred metagenome in this study showed that the PPAR signaling pathway, the insulin signaling pathway, and the adipocytokine signaling pathway in GDM2 patients were significantly decreased, suggesting that the induction of remission could partially restore the homeostasis of metabolic function. In particular, our study suggested that PPAR was positively correlated with the relative abundance of *Faecalibacterium* and negatively correlated with *Blautia*. Many literatures have reported that fatty acids can increase insulin sensitivity through the PPAR signaling pathway, thereby regulating blood glucose levels, which was closely related to the occurrence of GDM [68–70]. In our study, there is a positive correlation between *Faecalibacterium* in stool and blood glucose levels, while *Blautia* is the opposite. *Faecalibacterium* in GDM2 patients who can produce fatty acids (such as butyric acid and propionic acid) was significantly lower than that in N and GDM1. In conclusion, gut bacteria may participate in the pathological development of GDM2 through the PPAR signaling pathway, which should be conducted for further study in the future.

Besides the sample size, the other limitation of our study was that we only analyzed one stool sample per participant and was collected at 24-28 weeks of pregnancy. However, we are unable to clarify the causal relationship between the microbiome and the development of GDM due to a cross-sectional design. Consequently, data acquisition at multiple time points assists in providing further insights into their dynamic relationship. To confirm the associations observed in the current study, a large prospective cohort investigation and analysis of other potentially significant variables are necessary.

## 5. Conclusion

*Blautia*, *Faecalibacterium*, and *Eubacterium_hallii_group* had important characteristic changes in the gut microbiota of women with GDM2. The relative abundance of *Faecalibacterium*, *Blautia*, and *Eubacterium_hallii_group* can discriminate GDM2 from GDM. Gut bacteria may participate in the pathological development of GDM2 through the PPAR signaling pathway, which should be conducted for further study in the future.

## Figures and Tables

**Table 1:** Characteristics of the study population.

Descriptive measurements	N ($\overset{¯}{X}\pm \text{SD}$)	GDM1 ($\overset{¯}{X}\pm \text{SD}$)	GDM2 ($\overset{¯}{X}\pm \text{SD}$)	One-way ANOVA		ANOVA-linear term trend analysis	
				*F*	*P*	*F*	*P*
Age (years)	30.8 ± 4.8	34.3 ± 3.8	33.6 ± 5.6	2.8	0.07	2.529	0.118
Gestational week (weeks)	25.9 ± 1.1	26.0 ± 1.1	25.2 ± 0.9	2.1	0.14	2.689	0.107
Body weight (kg)	64 ± 9	65 ± 11	71 ± 12	1.8	0.18	3.090	0.085
Height (cm)	162 ± 3	162 ± 4	162 ± 5	0.08	0.93	0.058	0.811
BMI	24.3 ± 2.9	24.9 ± 3.8	27.2 ± 3.8	2.5	0.09	4.547	0.037
Abdominal circumference (cm)	90 ± 8	91 ± 7	97 ± 9	3.3	0.05	5.879	0.019
Systolic BP (mmHg)	121.8 ± 12.3	118.4 ± 10.3	120.6 ± 9.1	0.5	0.6	0.081	0.777
Diastolic BP (mmHg)	70.06 ± 11.6	69.88 ± 9.2	71.08 ± 17.8	0.04	0.96	0.047	0.829
Biochemistry							
FBG (mmol/L)	4.3 ± 0.3	4.7 ± 0.5	6.0 ± 0.6	49.9	<0.001	92.57	<0.001
1-hour blood glucose post 75 g oral glucose load (mmol/L)	7.0 ± 1.3	10.7 ± 1.5	12.3 ± 2.5	37.8	<0.001	67.06	<0.001
2-hour blood glucose post 75 g oral glucose load (mmol/L)	5.8 ± 0.9	8.9 ± 1.4	10.0 ± 2.0	32.5	<0.001	55.36	<0.001
TG (mmol/L)	2.2 ± 0.4	2.1 ± 0.6	2.6 ± 1.0	3.4	0.4	3.922	0.053
TC (mmol/L)	5.5 ± 1.1	6.8 ± 1.3	5.6 ± 0.9	7.9	0.001	0.006	0.939
HDL-C (mmol/L)	1.7 ± 0.4	2.1 ± 0.4	1.7 ± 0.3	4.5	0.016	0.003	0.954
LDL-C (mmol/L)	2.5 ± 0.9	3.3 ± 0.9	2.4 ± 0.7	7.3	0.002	0.117	0.734

## Data Availability

The data used to support the findings of this study are available from the corresponding authors upon request.

## Abbreviations

GDM: Gestational diabetes mellitus
FIGO: The International Federation of Gynecology and Obstetrics
LEfSe: Linear discriminant analysis effect size
OGTT: Oral glucose tolerance test
OTU: Operational taxonomic unit
rRNA: Ribosomal RNA
FBG: Fasting blood glucose.

## Supplementary Materials

Supplementary Materials Fig S1: rarefaction curve was used to evaluate the richness of fecal bacteria in all groups (at the level of 97% similarity). Fig S2: gut microbiota differences among the N, the GDM1, and the GDM2 groups (a) represents the phylum level, (b) represents the class level, (c) represents the order level, and (d) represents the family level. Pairwise comparison in multiple groups was conducted with Benjamin Hochberg, ^∗^*P* < 0.05, and ^∗∗^*P* < 0.01. Fig S3: taxonomic biomarkers between N and GDM2 LEfSe analysis shows differentially abundant genera as biomarkers determined using the Kruskal-Wallis test (*P* < 0.05) with LDA score > 4.0. The LDA discriminant histogram statistics showed that there were significant microbial groups in the two groups. The LDA score was obtained by LDA (linear regression analysis); the larger the LDA score, the greater the influence of species abundance on the difference effect. Fig S4: the predicted functional module involving PPAR signaling pathways among N, GDM1, and GDM2. (a) Insulin signaling pathway; (b) adipocytokine signaling pathway; (c) progesterone-mediated oocyte maturation. Table S1: suggestions of the dietary examples (1800 kcal). Table S2: suggestions of dietary examples (2000 kcal). Table S3: bacterial alpha diversity estimators. Table S4: the relative abundance of gut microbiota (genus level) among N, GDM1, and GDM2. Table S5: potential trends in the relative abundance of gut microbiota among the groups with Spearman's rank correlation test.
