# Supplementary material for: The Gut Microbiota in Women Suffering from Gestational Diabetes Mellitus with the Failure of Glycemic Control by Lifestyle Modification
Source: J Diabetes Res. 2019 Oct 23;2019:6081248. doi: 10.1155/2019/6081248 (PMC6854930; doi:10.1155/2019/6081248)
Supplement: Supplementary Materials — Fig S1: rarefaction curve was used to evaluate the richness of fecal bacteria in all groups (at the level of 97% similarity). Fig S2: gut microbiota differences among the N, the GDM1, and the GDM2 groups (a) represents the phylum level, (b) represents the class level, (c) represents the order level, and (d) represents the family level. Pairwise comparison in multiple groups was conducted with Benjamin Hochberg, ∗P < 0.05, and ∗∗P < 0.01. Fig S3: taxonomic biomarkers between N and GDM2 LEfSe analysis shows differentially abundant genera as biomarkers determined using the Kruskal-Wallis test (P < 0.05) with LDA score > 4.0. The LDA discriminant histogram statistics showed that there were significant microbial groups in the two groups. The LDA score was obtained by LDA (linear regression analysis); the larger the LDA score, the greater the influence of species abundance on the difference effect. Fig S4: the predicted functional module involving PPAR signaling pathways among N, GDM1, and GDM2. (a) Insulin signaling pathway; (b) adipocytokine signaling pathway; (c) progesterone-mediated oocyte maturation. Table S1: suggestions of the dietary examples (1800 kcal). Table S2: suggestions of dietary examples (2000 kcal). Table S3: bacterial alpha diversity estimators. Table S4: the relative abundance of gut microbiota (genus level) among N, GDM1, and GDM2. Table S5: potential trends in the relative abundance of gut microbiota among the groups with Spearman's rank correlation test. [file 6081248.f1.pdf]

Supplementary material

The gut microbiota in women suffering from gestational diabetes mellitus with the failure of glycemic control by lifestyle modification

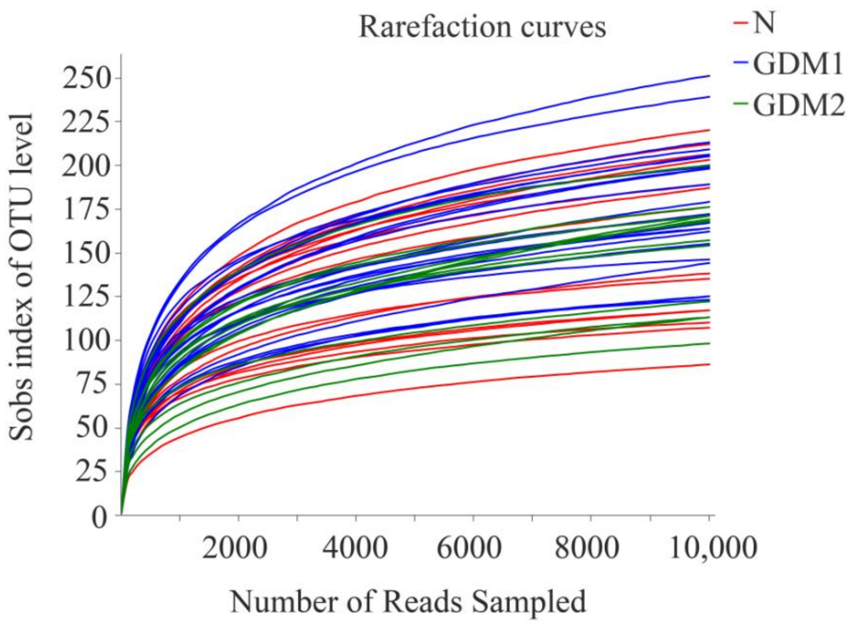

Fig S1:Rarefaction curve was used to evaluate the richness of fecal bacteria in all groups (at the level of 97% similarity)

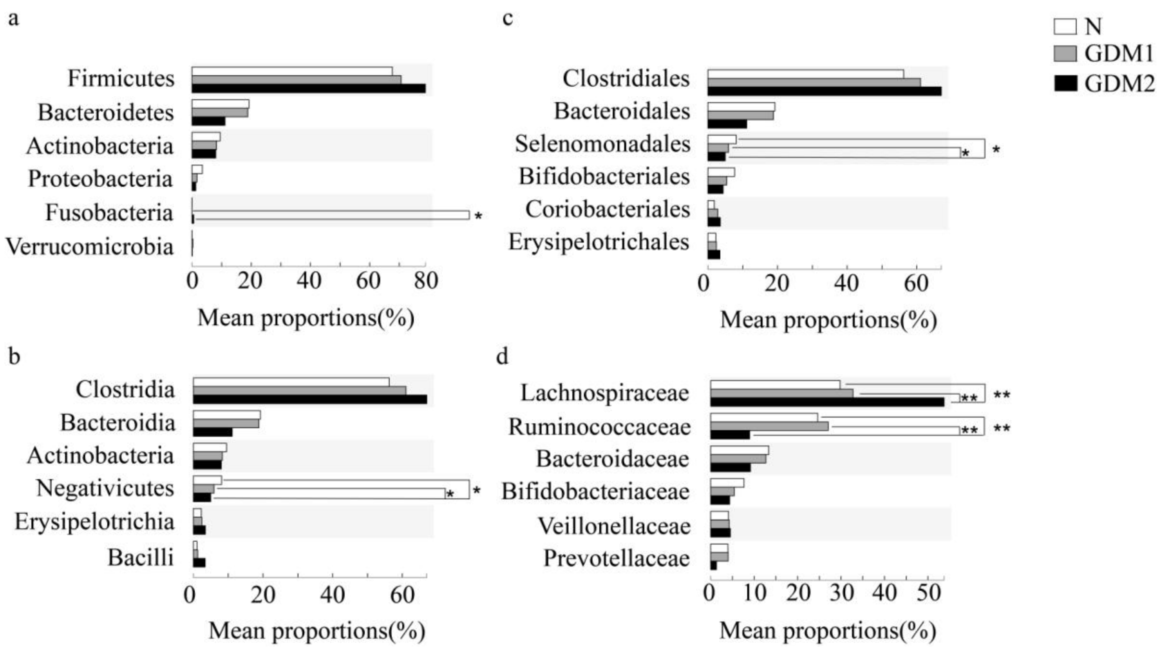

Fig S2: Gut microbiota differences among the N, GDM1, and GDM2 groups

a represents the phylum level, **b** represents the class level, **c** represents the order level, and **d** represents the family level. Pairwise comparison in multiple groups was conducted with Benjamin Hochberg, *$P < 0.05$, and **$P < 0.01$.

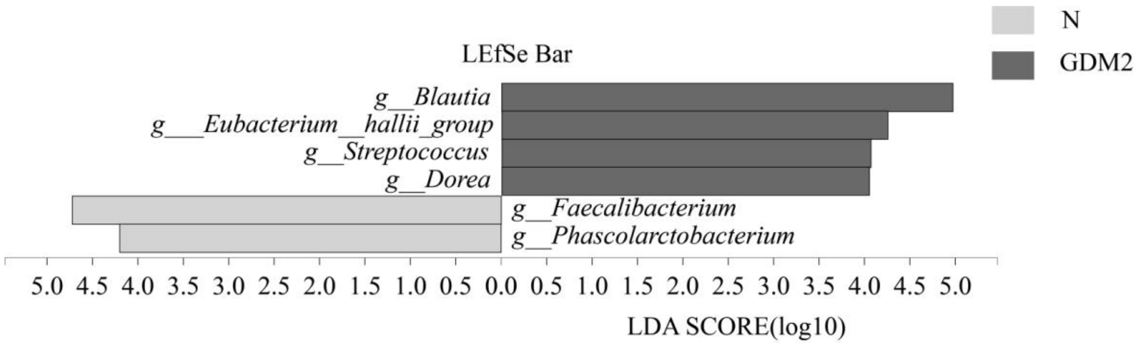

Fig S3: Taxonomic biomarkers between N and GDM2

LEfSe analysis shows differentially abundant genera as biomarkers determined using Kruskal-Wallis test ($P < 0.05$) with LDA score > 4.0. The LDA discriminant histogram statistics showed that there were significant microbial groups in the two groups. The LDA score obtained by LDA analysis (linear regression analysis), the larger the LDA score, the greater the influence of species abundance on the difference effect.

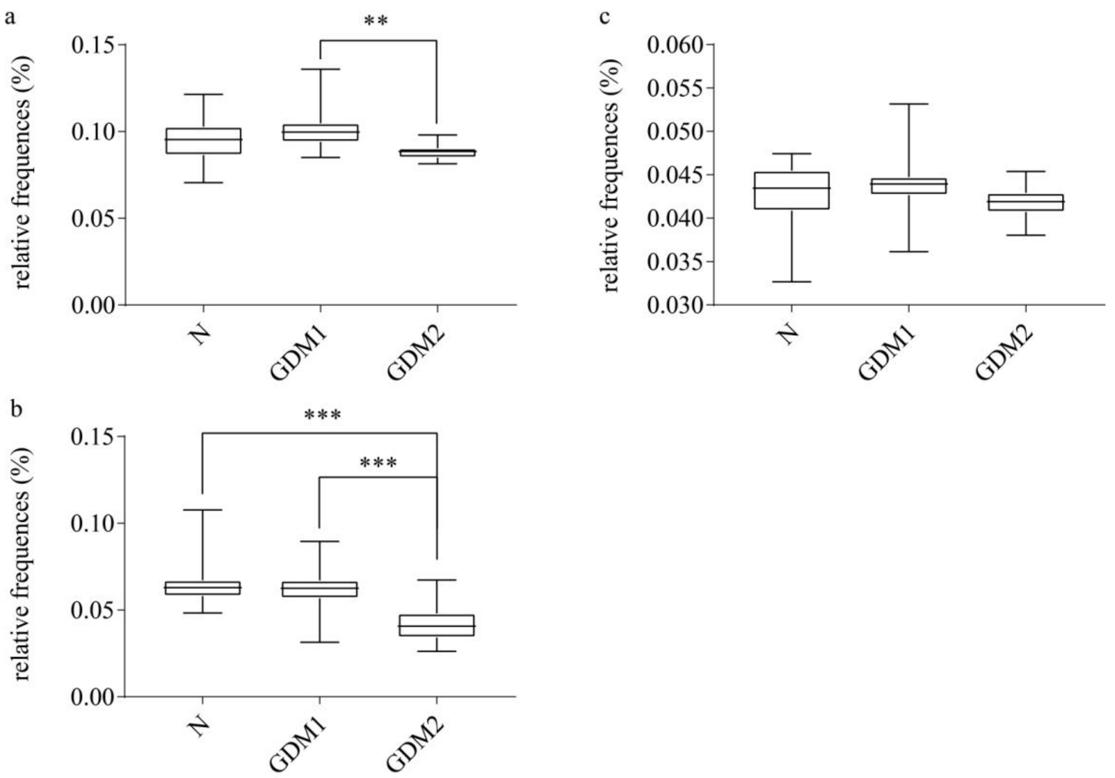

Fig S4: The predicted functional module involving PPAR signaling pathways among N, GDM1 and GDM2

a. Insulin signaling pathway; b. Adipocytokine signaling pathway; c. Progesterone mediated oocyte maturation.

Table S1. Suggestions of the dietary examples (1800 kcal)

Meals	The food and the amount	Notes
Breakfast 7:00-8:00	1 staple food (e.g. 1 whole-wheat bread, 35g)	about 300 kcal
	cold melon and fruit 150g (e.g. cucumber and tomato or 150g lettuce)	
	1 boiled egg (60g)	
	1 low fat milk (200ml)	
Snacks 9:00-10:00	Choose two from the following three choices:	about 180-200 kcal
	1 fruit (about 200g)	
	1 nut (e.g. 35g walnut or 15g cashew)	
	Yi Lijia (3 spoons)	
Lunch 12:00	a small bowl of coarse cereals (75g)	about 550 kcal (3g salt; 10g vegetable oil)
	1 meat food (e.g. 50g pig platoon or beef, or 100g fish and shrimp)	
	50-100g old tofu or dried tofu	
	At least 3 kinds of seasonal vegetables for about 200-250g	
Snacks 14:00-15:00	1 cereals and potato food (e.g. 1 corn cob or 100g taro or 3 spoons Yi Lijia)	about 135 kcal
	Half fruit (about 100g)	
Dinner 17:00-18:00	a small bowl of coarse cereals (75g)	about 550 kcal (3g salt; 10g vegetable oil)
	2 copies fish and meat (e.g. 200g fish or 150g chicken)	
	At least 3 kinds of seasonal vegetables for about 200-250g	
Snacks 20:00-21:00	Choose one from the following two choices:	about 90-110 kcal
	130g sugar-free yogurt or 1 dessert for about 90g Yi Lijia (3 spoons)	

According to blood glucose and weight, exercise should be performed for 30 to 40 minutes after meal

Table S2. Suggestions of dietary examples (2000 kcal)

Meals	The food and the amount	Notes
Breakfast 7:00-8:00	1 staple food (e.g. 1 whole-wheat bread, 35g)	about 300 kcal
	cold melon and fruit 150g (e.g. cucumber and tomato or 150g lettuce)	
	1 boiled egg (60g)	
	1 low fat milk (200ml)	
Snacks 9:00-10:00	Choose two from the following three choices:	about 180-200 kcal
	1 fruit (e.g. 200g apples or pears, and 150g bananas)	
	1 nut (e.g. 35g walnut or 15g cashew)	
	Yi Lijia (3 spoons)	
Lunch 12:00	a small bowl of coarse cereals (75g)	about 550 kcal (3g salt; 10g vegetable oil)
	1 meat food (e.g. 50g pig platoon or beef, or 100g fish and shrimp)	
	50-100g old tofu or dried tofu	
	At least 3 kinds of seasonal vegetables for about 200-250g	
Snacks 14:00-15:00	1 cereals and potato food (e.g. 1 corn cob or 100g taro or 3 spoons Yi Lijia)	About 200 kcal
	Half fruit (about 100g)	
Dinner 17:00-18:00	a small bowl of coarse cereals (75g)	about 550 kcal (3g salt; 10g vegetable oil)
	2 copies fish and meat (e.g. 200g fish or 150g chicken)	
	At least 3 kinds of seasonal vegetables for about 200-250g	
Snacks 20:00-21:00	Choose one from the following two choices:	about 180 kcal
	200g low fat milk	
	25g sugar-free oatmeal	

Yi Lijia (3 spoons)
According to blood glucose and weight, and take exercise for 30 to 40 minutes after meal

Table S3. Bacterial alpha diversity estimators

	N ($\overline{X}$ ±SD)	GDM1 ($\overline{X}$ ±SD)	GDM2 ($\overline{X}$ ±SD)	*P*
Sobs	161.88±44.66	177.42±34.19	151±31.654	0.36
chao	192.49±56.74	218.04±42.96	194.78±43.61	0.26
shannon	3.47±0.43	3.53±0.41	3.41±0.41	0.66
simpson	0.07±0.04	0.07±0.06	0.07±0.04	0.80
shannoneven	0.69±0.06	0.68±0.07	0.68±0.06	0.96
simpsoneven	0.11±0.04	0.10±0.04	0.11±0.04	0.58

Note: The Kruskal-Wallis H test was used for comparing three groups.

Table S4. The relative abundance of gut microbiota (genus level) among N, GDM1 and GDM2

	N	GDM1	GDM2	Chi-Square	*P*
Blautia	0.049 (0.045)	0.068(0.031)	0.255 (0.213)	16.368	2.791E-4
Faecalibacterium	0.136(0.106)	0.129(0.139)	0.044(0.055)	11.640	2.968E-3
Eubacterium_hallii_group	0.009(0.009)	0.012 (0.012)	0.049(0.041)	20.123	4.270E-5
Subdoligranulum	0.033(0.040)	0.043 (0.057)	0.015 (0.019)	8.270	0.016
Phascolarctobacterium	0.010(0.045)	0.011(0.021)	0.002(0.002)	7.276	0.026
Roseburia	0.024 (0.019)	0.014(0.023)	0.006 (0.009)	6.844	0.033

Note: The Kruskal-Wallis H test was used for comparing three groups. Data were presented as median (interquartile range).

Table S5. Potential trends in the relative abundance of gut microbiota among the groups with Spearman's rank correlation test

	Trends in the relative abundance among the groups[a]	
	r	*P*
Blautia	0.533	0.000048
Eubacterium_hallii_group	0.599	0.000003
Faecalibacterium	-0.419	0.002

Note: a: N (Assignment: 0), GDM1 (Assignment: 1), and GDM2 (Assignment: 2)
